# Predictive Hypoxemic Threshold for Tolerating the Apnea Test While Assessing Death by Neurological Criteria

**DOI:** 10.1007/s12028-024-02105-z

**Published:** 2024-09-19

**Authors:** Daniel Aviram, Daniel Hikri, Michal Aharon, Amir Galoz, Yael Lichter, Noam Goder, Asaph Nini, Nimrod Adi, Dekel Stavi

**Affiliations:** 1 Division of Anesthesia, Pain Management and Intensive Care, Tel Aviv Sourasky Medical Center, Tel Aviv, Israel; 2 Faculty of Medicine, Tel Aviv University, Tel Aviv, Israel; 3 Organ Transplantation Unit, Tel Aviv Sourasky Medical Center, Tel Aviv, Israel; 4 Critical Care Unit, University College Hospital, London, UK

**Keywords:** Brain death, Organ donation

## Abstract

**Background:**

The apnea test (AT) plays a vital role in diagnosing brain death by evaluating the absence of spontaneous respiratory activity. It entails disconnecting the patient from mechanical ventilation to raise the CO_2_ partial pressure and lower the pH. Occasionally, the AT is aborted because of safety concerns, such as hypoxemia and hemodynamic instability, to prevent worsening conditions. However, the exact oxygen partial pressure level needed before commencing AT, indicating an inability to tolerate the test, is still uncertain. This study seeks to determine pre-AT oxygen levels linked with a heightened risk of test failure.

**Methods:**

We conducted a retrospective cohort study involving patients suspected of having brain death at the Tel Aviv Medical Center from 2010 to 2022. The primary outcome was defined as an arterial partial O_2_ pressure (PaO_2_) level of 60 mmHg or lower at the conclusion of the AT. This threshold is significant because it marks the point at which the saturation curve deflects, potentially leading to rapid deterioration in the patient’s oxygen saturation.

**Results:**

Among the 70 patients who underwent AT, 7 patients met the primary diagnostic criteria. Patients with a PaO_2_ ≤ 60 mmHg at the conclusion of the AT exhibited a significantly lower initial median PaO_2_ of 243.7 mmHg compared with those with higher pre-AT PaO_2_ levels of 374.8 mmHg (interquartile range 104.65–307.00 and interquartile range 267.8–444.9 respectively, *P* value = 0.0041). Pre-AT PaO_2_ levels demonstrated good discriminatory ability for low PaO_2_ levels according to the receiver operating characteristic (ROC) curve, with an area under the curve of 0.76 (95% confidence interval 0.52–0.99).

**Conclusions:**

PaO_2_ values at the conclusion of the AT are closely associated with PaO_2_ values at the beginning of the test. Establishing a cutoff value of approximately 300 mmHg PaO_2_ at the onset of AT may assist in avoiding saturation drops below 90%.

## Introduction

Analyzing the diagnosis of brain death entails two principal considerations. The first revolves around the intricate question of determining whether the patient is alive or deceased, posing an ethical quandary at the end of life in cases of irreversible coma. The second pertains to organ donation, given the continually rising demand for transplantation [1].

The American Academy of Neurology (AAN) has established practical parameters for diagnosing brain death or death by neurological criteria (DNC), which serve as the basis for numerous global diagnostic protocols [2]. The 2023 update of the AAN guidelines provides unified specifications, stating that DNC should be declared when a patient with a known cause of catastrophic brain injury exhibits permanent loss of function across the entire brain, including the brainstem. This is evidenced by coma, brainstem areflexia, and apnea in response to an appropriate stimulus [3].

With no published reports of recovery of neurologic function after a diagnosis of brain death using these criteria [4], this diagnosis indicates the complete absence of recovery possibilities and enables the option for organ donation in accordance with the “dead donor rule” [5].

The apneic oxygen diffusion test, or apnea test (AT), is a critical component in the diagnosis of DNC [6]. It involves temporarily disconnecting the patient from mechanical ventilation while providing oxygen flow to evaluate the capacity for independent breathing. In the absence of any other alternative explanation, the lack of respiratory effort when arterial partial CO_2_ pressure (PaCO_2_) exceeds 60 mmHg and is at least 20 mmHg above the baseline level, provides compelling evidence of brainstem dysfunction and indicate DNC [7].

Globally, AT has become pivotal in assessing DNC [8]. Nevertheless, despite its widespread application, AT poses risks, primarily linked to exacerbating hypoxia, hypotension, arrhythmias, and even cardiac arrest [9]. Consequently, guidelines advise predefined safety thresholds, prompting test termination [6].

When AT is aborted, it often prompts a decision to retry after optimizing the patient’s condition or resorting to ancillary tests [10]. Given the emotional toll of losing a family member, prolonged and unexpected testing may induce family confusion, frustration, mistrust, and, in some instances, objections to the process.

The reported incidence of aborted AT ranges from 0 to 20%, with hypoxemia being a key contributing factor [11]. According to AAN guidelines, maintaining a preoxygenation PaO_2_ above 200 mmHg before AT is recommended [6]. However, the specific initial hypoxia levels at the pre-AT stage, indicating intolerance to the test, remain unclear and warrant further investigation. Our goal in this study was to explore the predictive value of PaO_2_ levels at the preoxygenation stage that could be addressed as a cutoff for successful process and the levels at which AT failure is at higher risk.

## Methods

All patients between the age of 18–80 years old assessed for brain death between 2010 and 2022 at the Tel Aviv Sourasky Medical Center were reviewed. Data including demographics, background illness, type of injury, suspected cause of brain death, level of mechanical ventilation support, arterial blood gas analysis before AT was commenced and at the end of the process, and completion of AT were collected from the electronic medical records.

A PaO_2_ value of 60 mmHg at the end of AT was defined as the cutoff value as saturation of 90% by pulse oximeter (roughly PaO_2_ of 60 mmHg) is set as a safety lower limit, according to the national directive, and requires abortion of the AT. This PaO_2_ value at the end of the AT was then plotted against PaO_2_ values prior to AT initiation (PaO_2_/FiO_2_ ratio for all the patients equals their PaO_2_ because all patients were given 100% oxygen at that point) in search of a correlation.

### AT Protocol

In our institute, AT is performed according to the national directives of brain death diagnosis and the AAN guidelines [6].

Patients suspected of having brain death were provided mechanical ventilation via an endotracheal tube, whereas invasive blood pressure monitoring was established using an arterial line. Prior to commencing AT, specific baseline parameters were ensured according to the national guidelines: body temperature above 34 °C, systolic blood pressure above 90 mmHg, pH levels within the range of 7.35–7.45, and PaCO_2_ between 35 and 45 mmHg. Patients were put on vasopressor support as needed to keep with hemodynamic goals. Two certified physicians, authorized by the Ministry of Health to determine neurological unresponsiveness, conducted neurological examinations before initiating the AT. Each AT session began after a minimum of 10 min of preoxygenation with 100% FiO_2_ and following the optimization of positive end-expiratory pressure (PEEP) to attain the highest achievable pulse saturation. The same PEEP values were kept using a valve placed on the inflating resuscitation bag throughout the test. Arterial blood gases were analyzed as a baseline. During the AT, 100% oxygen was administered via a flow-inflating resuscitation bag with a PEEP valve, maintaining the same PEEP level as before disconnection. Criteria for terminating the AT included respiratory effort, occurrence of arrhythmias, pulse oximetry saturation dropping below 90%, or systolic blood pressure declining below 90 mmHg for more than 30 s. If none of these events occurred within 10 min, an arterial blood gas value was obtained. The AT concluded if the PaCO_2_ exceeded 60 mmHg and was at least 20 mmHg higher than the baseline.

### Statistical Analysis

Continuous variables were summarized using medians and interquartile ranges, with intergroup comparisons conducted using the Mann–Whitney *U*-test. For categorical variables, counts and percentages were employed for summarization, and group comparisons were made using the Fisher exact test.

To assess the discriminative ability of PaO_2_ values at the start as potential predictors for desaturation, a ROC model was applied. This model was instrumental in determining the optimal threshold for identifying desaturation.

Furthermore, a logistic regression model was employed to ascertain factors influencing the odds of desaturation, with adjustments made for age and sex.

The significance level was set at *P* < 0.05, employing a two-sided test. All analyses were conducted using R version 4.3.2 (R Foundation for Statistical Computing, Vienna, Austria).

## Results

A total of 100 patients were assessed for the diagnosis of DNC between the years 2010 and 2022, of whom 7 were under the age of 18, eighteen had not performed AT, and 5 had insufficient electronic record documentation, giving a total of 70 patients who were included in our analysis. All patients met the preliminary AAN criteria from 2010, including systolic blood pressure of 100 mmHg or above and a core body temperature of at least 36 °C even though our national guidelines allow lower values (i.e., a systolic blood pressure of 90 mmHg and a core temperature of 34 °C).

Of the study population, 27 (38.6%) were female, and the mean age was 53.11 years (standard deviation [SD] of 15.44 years).

In terms of other comorbidities, 22 patients (31.4%) had a diagnosis of hypertension, 14 patients (20.0%) had diabetes, and 1 patient (1.4%) had a diagnosis of heart failure. None of the patients had a diagnosis of liver cirrhosis, peripheral vascular disease, or chronic renal failure. The leading etiologies for brain injury were spontaneous intracranial hemorrhage, anoxic brain injury, and trauma (38.6%, 34.3%, and 15.7%, respectively).

Of the 70 patients, 7 (10%) had a PaO_2_ ≤ 60 mmHg at the end of the AT and were defined as group A, and 63 (90%) patients had a PaO_2_ > 60 mmHg at the end of the AT and were defined as group B. In five patients, DNC was not concluded because of AT abortion: three of five aborted tests were due to hypoxemia (i.e., reaching an SpO_2_ lower than 90%, which is the defined safety limit according to our national guidelines), and the other two tests were aborted due to hemodynamic instability. These five patients were included in our analysis. The total mean AT time was 9.51 min (SD of 2.4), with a mean of 5.14 min (SD 3.58) for group A and 10.03 min (SD 1.64) for group B. None of the patients required nitric oxide inhalation prior to the AT.

No statistically significant differences in demographics, background illness, or type of injury were found between the two groups, as depicted in Table 1. No statistically significant difference in systolic blood pressure, core temperature, or PEEP at the beginning and end of AT was found between the two groups (Table 2). No significant changes between the groups were seen in term of pulmonary comorbidity: known chronic lung disease (*P* value = 0.44), lung infection (*P* value = 0.628), acute respiratory distress syndrome (*P* value = 0.735), and pulmonary embolism (*P* value = 0.735).

**Table 1:** Comparative analysis of demographics and clinical characteristics between groups A and B

	Group A after AT PaO_2_ ≤ 60 (*n* = 7)	Group B after AT PaO_2_ > 60 (*n* = 63)	*P* value
Female, *n* (%)	2 (28.6)	25 (39.7)	0.57
Age, mean (SD) (yr)	47.57 (21.72)	53.73 (14.69)	0.46
Known chronic lung disease, *n* (%)	1 (14.3)	4 (6.3)	0.44
Hypertension	2 (28.6)	20 (31.7)	0.86
Diabetes	2 (28.6)	12 (19.0)	0.55
Heart failure	0 (0.0)	1 (1.6)	0.74
Previous stroke	1 (14.3)	5 (7.9)	0.57
Chronic kidney failure	0 (0.0)	0 (0.0)	1
Steroids use	7 (100.0)	58 (92.1)	0.44
Eltroxin therapy	4 (57.1)	27 (42.9)	0.47
Active lung infection	1 (14.3)	14 (22.2)	0.63
ARDS	0 (0)	1 (1.6)	0.74
Pulmonary embolism	0 (0)	1 (1.6)	0.74
Etiology			
Trauma	1 (14.3)	10 (15.9)	
Spontaneous intracranial hemorrhage	2 (28.6)	25 (39.7)	
Anoxic brain injury	3 (42.9)	21 (33.3)	
Brain tumor	0 (0.0)	0 (0.0)	
Stroke	0 (0.0)	5 (7.9)	
Brain edema	1 (14.3)	2 (3.2)	

ARDS, acute respiratory distress syndrome, AT, apnea test, SD, standard deviation

**Table 2:** Comparative analysis of demographics and clinical characteristics between groups A and B

	Group A after AT PaO_2_ ≤ 60 (*n* = 7)	Group B after AT PaO_2_ > 60 (*n* = 63)	*P* value
Completion of AT, *n* (%)	5 (71.4)	60 (95.2)	0.02
AT time, mean (SD) (min)	5.14 (3.58)	10.03 (1.64)	< 10 × 10^4^
pH at start of AT, mean (SD)	7.37 (0.05)	7.38 (0.07)	0.53
pH at end of AT, mean (SD)	7.26 (0.04)	7.18 (0.06)	0.001
Temp at start of AT, mean (SD)	37.03 (0.65) °C	36.12 (4.81) °C	0.169
Temp at end of AT, mean (SD)	37.03 (0.65) °C	36.75 (0.92) °C	0.329
SBP at start of AT, mean (SD)	145.43 (31.13) mmHg	140.03 (35.35) mmHg	0.429
SBP at end of AT, mean (SD)	133.86 (37.34) mmHg	142.47 (33.84) mmHg	0.579
PEEP, mean (SD)	5.57 (0.98) CmH_2_O	5.4 (1.32) CmH_2_O	0.688
PCO2 at start of AT, mean (SD)	45.27 (4.01) mmHg	46.28 (6.59) mmHg	0.572
PCO_2_ at end of AT, mean (SD)	61.76 (12.73) mmHg	80.16 (15.38) mmHg	0.01
PaO_2_ at start of AT, mean (SD)	230.13 (145.60) mmHg	360.70 (116.58) mmHg	0.0276
PaO_2_ at end of AT, mean (SD)	53.74 (4.41) mmHg	221.93 (120.13) mmHg	< 10 × 10^4^

AT, apnea test, PEEP, positive-end expiratory pressure, SBP, systolic blood pressure, SD, standard deviation, Temp, temperature

**Fig. 1:**
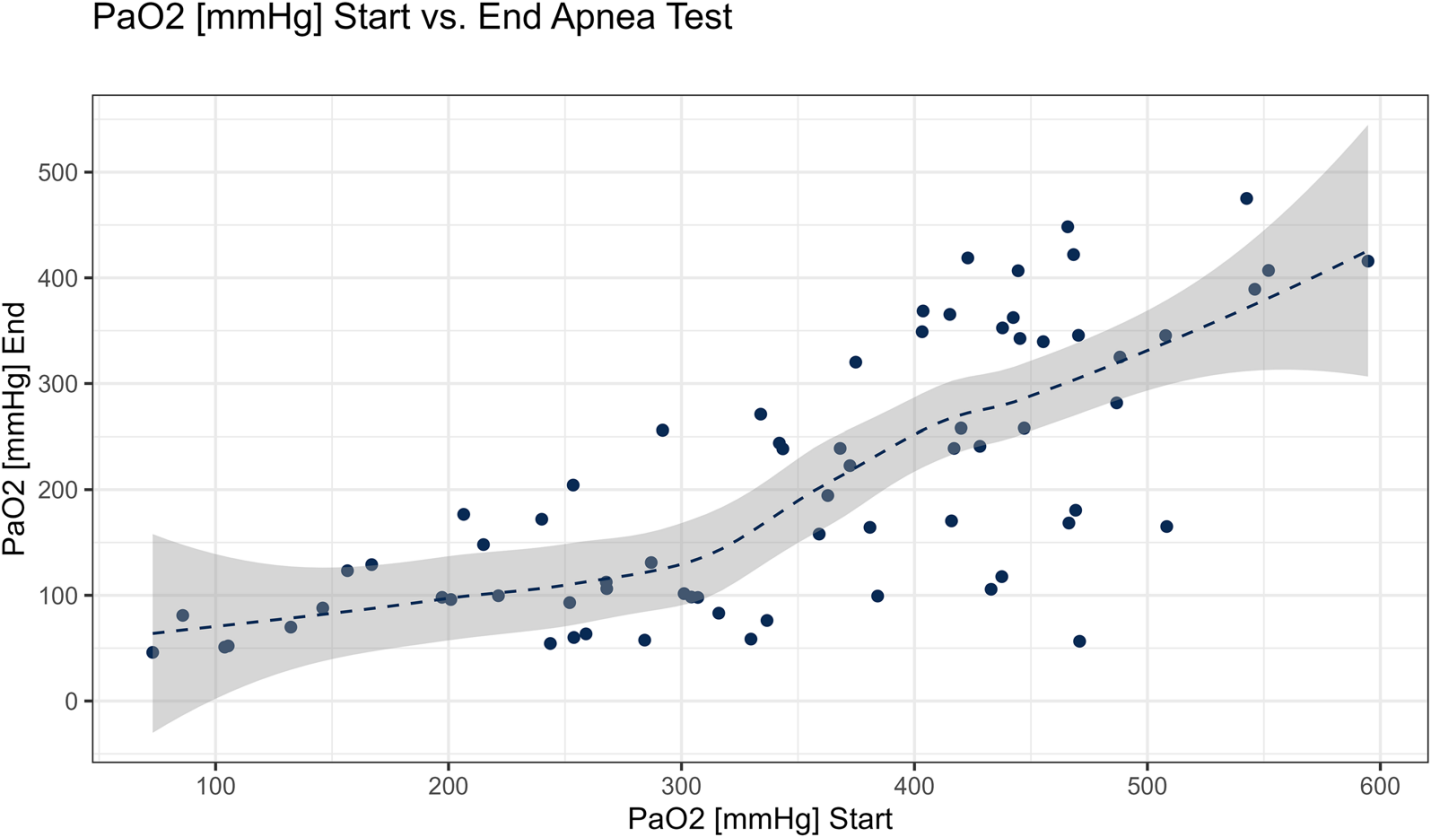
Relation of PaO_2_ at the beginning of the AT and the end. AT, apnea test

Higher PaO_2_ levels at the beginning of the AT corelated with higher PaO_2_ levels at the end of AT, as seen in Fig. 1. Of seven patients in total who reached the threshold of 60 mmHg, four patients had initial PaO_2_ values higher than 200 mmHg before AT begun. Three more patients were below 70 mmHg at the end of AT but did not cross the 60-mmHg limit. PCO_2_ levels at the end of the AT were significantly higher in group B (*P* value = 0.001), despite no significant difference on PCO_2_ levels at the beginning of AT, and these differences were also seen in pH values.

**Fig. 2:**
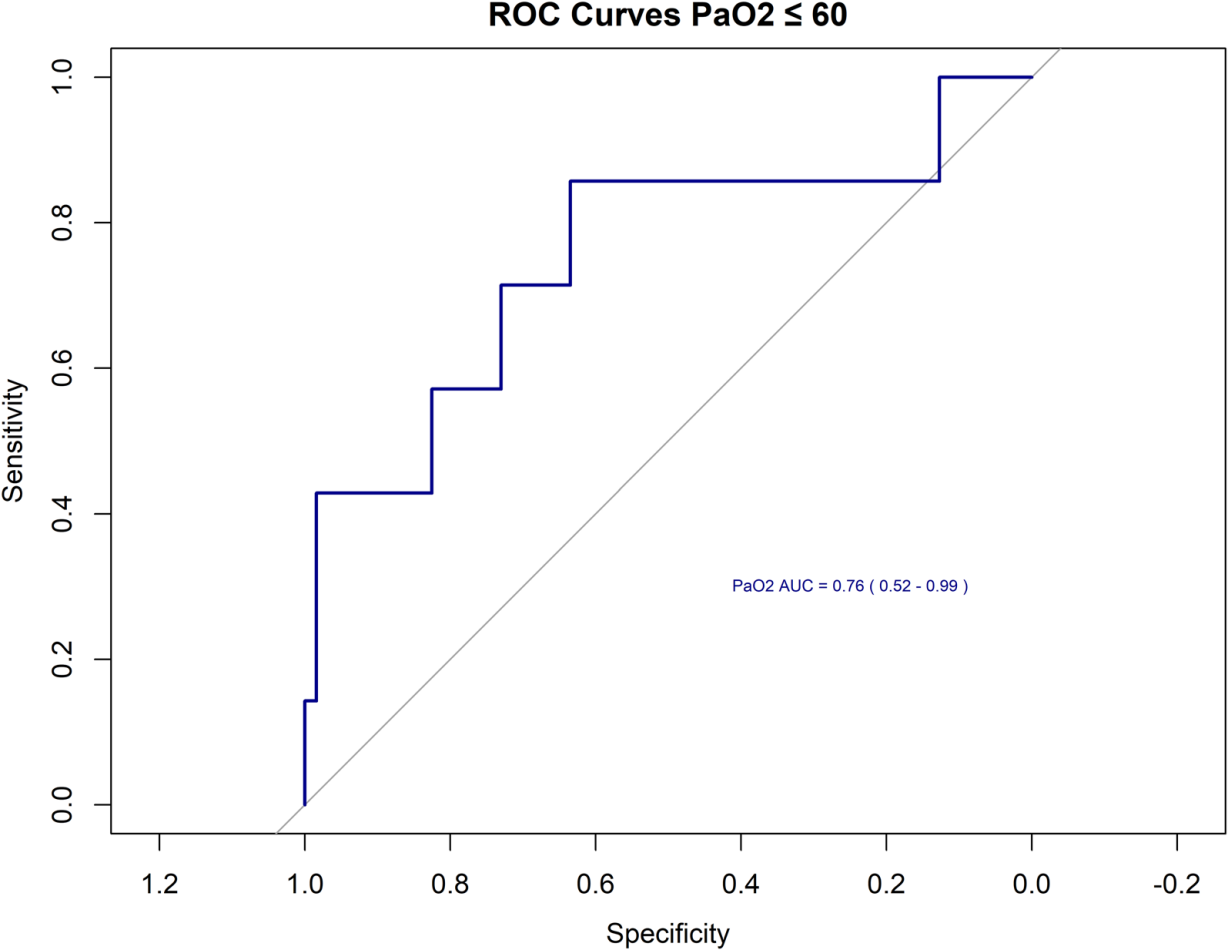
A receiver operating characteristic model for the threshold of PaO_2_ mmHg at the end of AT. AT, apnea test

As shown by the ROC curve analysis (Fig. 2), PaO_2_ levels before initiating AT had a well-discriminative ability to lower PaO_2_ levels at the end of the test, with an area under the curve of 0.76 (95% confidence interval [CI] 0.52–0.99). A threshold of a PaO_2_ of 300 mmHg at the start yielded a sensitivity of 71% and a specificity of 70%. A threshold of PaO_2_ of 330 mmHg at start yielded a sensitivity of 86% and a specificity of 63%.

**Fig. 3:**
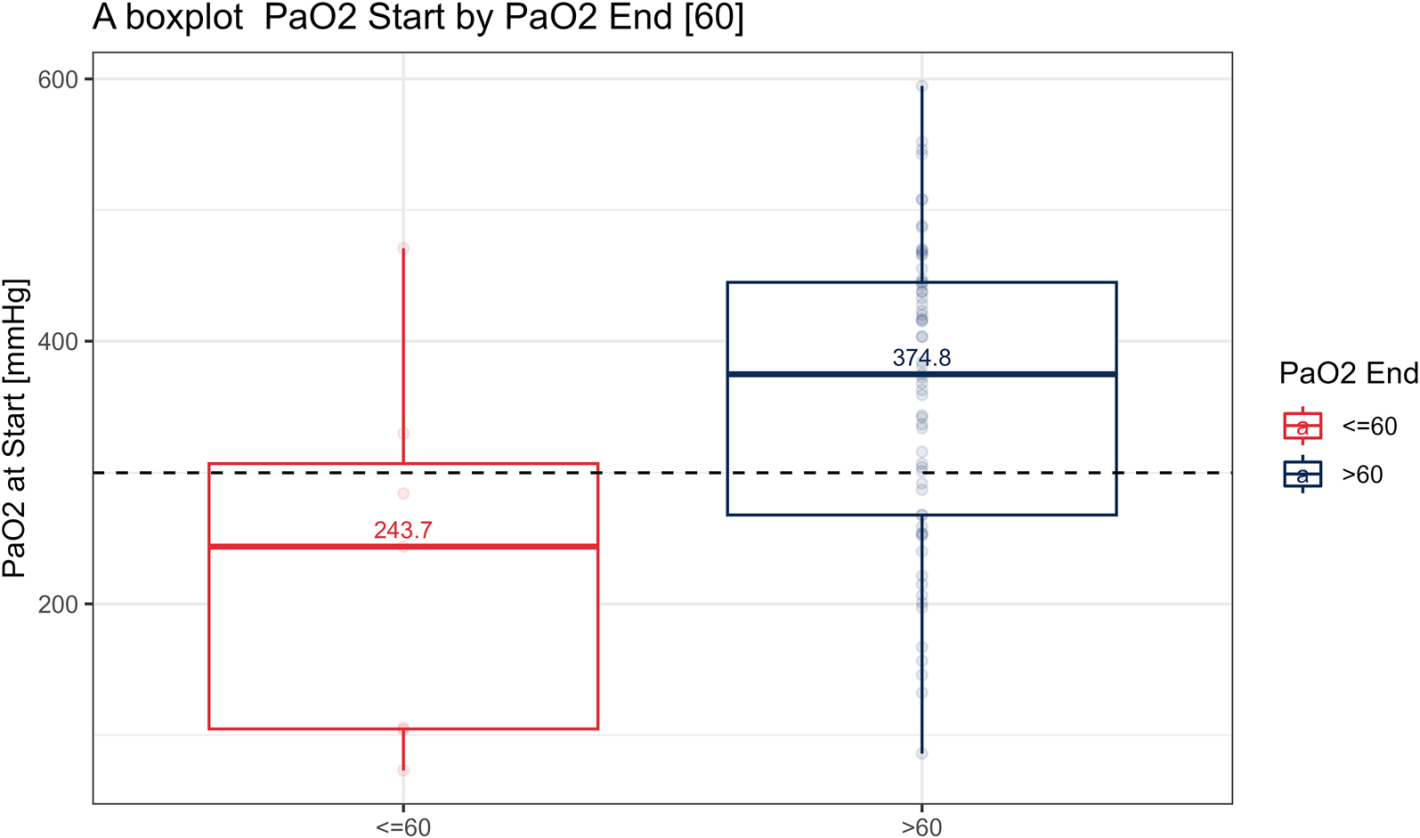
A boxplot of initial PaO_2_ of groups A and B

When comparing groups A and B, the median initial PaO_2_ was 243.7 mmHg for group A and 374.8 mmHg for group B (interquartile ranges of 104.65–307.00 and 267.8–444.9, respectively, yielding a *P* value of 0.0276; Fig. 3).

No statistically significant correlation was found after adjusting post-AT PaO_2_ levels < 60 mmHg using a logistic regression model for age (CI 0.9304–1.0520, *P* value 0.723) and sex (CI 0.1593–9.4694, *P* value of 0.931).

## Discussion

The apnea test, guided by AAN guidelines [6] and widely accepted globally, is integral to DNC assessment. Despite its common use, AT carries up to a 20% risk of patient deterioration, primarily linked to hypoxemia. Defining initial PaO_2_ levels that might predict a successful and safe AT are scarcely described and vary widely in the literature.

Complications during AT can lead to patient deterioration, AT abortion, and the need for subsequent attempts after stabilizing the patient’s condition or conducting additional tests. Besides the risks to the patient, such events can intensify the suffering of the patient’s family and close contacts. Therefore, assessing and mitigating risks for AT failure is crucial.

Because hypoxemia is a critical factor in the failure of AT, our study focused on identifying pre-AT oxygenation levels that could predict the success of the test, particularly in terms of meeting the oxygenation threshold for test termination. We examined the pre-AT PaO_2_ levels in patients (reflecting the PaO_2_/FiO_2_ ratio in mmHg due to 100% FiO_2_ administered) undergoing DNC assessments at our institution. Although the AAN guidelines suggest terminating the test if oxygen saturation falls below 85%, our national directives require test termination if arterial saturation remains below 90% for more than 30 s. Because there’s a strong linear correlation between PaO_2_ and oxygen saturation below 90%, and considering the likelihood of rapid oxygenation decline without ventilation on reaching this level, we chose a PaO_2_ of 60 mmHg at the end of the AT as a safety threshold to predict test outcomes.

The following observations could be drawn from this study’s results: (1) There is a correlation between the pre-AT PaO_2_ level and the PaO_2_ level at the conclusion of the AT. (2) Just over half of the patients (57%) with an end AT PaO_2_ ≤ 60 mmHg had an initial PaO_2_ exceeding 200 mmHg. (3) A cutoff for the initial PaO_2_ ranging from 278 to 307 mmHg could be discriminative, as 75% of the patients from group A (an end PaO_2_ lower than 60 mmHg) had an initial PaO_2_ value below the threshold of 307 mmHg, and 75% of the patients from group B (end PaO_2_ levels higher than 60 mmHg) had an initial PaO_2_ above 278 mmHg. (4) A higher initial PaO_2_ level of 300 mmHg and 330 mmHg would exhibit a higher sensitivity of 71% and 86%, respectively, in identifying patients who will not achieve the 60-mmHg threshold at the end of AT. However, specificity decreases accordingly, indicating patients who may not reach this level despite having a lower initial PaO_2_ level.

The AAN guidelines recommend a preoxygenation period of at least ten minutes with 100% oxygen and an initial PaO_2_ of 200 mmHg before commencing the AT for safety and successful assessment of thew AT. During the AT, 100% oxygen is required to be administered either via a flow-inflating resuscitation bag with a PEEP valve or through a catheter placed just above the carina [6]. It is not uncommon for patients in the process of declaring DNC, to suffer from suboptimal lung gas exchange capability resulting from trauma, mechanical ventilation, hemodynamic, and cardiac performances changes. In patients for whom the lung’s oxygenation capability is reduced and PaO_2_ levels before AT are not sufficient, measures to improve lung performance could include endobronchial secretions suctioning, recruitment maneuvers, and negative fluid balance.

Different studies reported different optimal thresholds for initial PaO_2_ levels before AT, and a wide variety of levels were suggested.

Yee et al.’s [12] study found no association between pre-AT PaO_2_/FiO_2_ ratio and test outcomes, but a median A-a gradient of 376 mmHg and 175 mmHg, equivalent to PaO_2_ of 287 and 488 respectively, (assuming FiO_2_ of 100% and PCO_2_ of 40 mmHg) was reported in the aborted AT group and the test completion group, respectively. In contrast, Kim’s et al. [13] study, which included 512 patients (42% of them reporting with moderate to severe acute respiratory distress syndrome), proposed an A-a gradient of 556.4 mmHg as the optimal cutoff for AT failure (equivalent to PaO_2_ of 107 mmHg with FiO_2_ of 100% and PCO_2_ of 40 mmHg).

Our research delved into the natural oxygenation kinetics during the pre-AT period and its progression. We observed a correlation between pre-AT PaO_2_ values and PaO_2_ values at the end of AT. Despite some patients having initially sufficient PaO_2_ levels, they reached the safety limit, leading to AT termination. Using these kinetics and establishing a cutoff of an initial PaO_2_ value of approximately 300 mmHg may aid in predicting the likelihood of reaching saturation limits, preventing severe hypoxemia and subsequent deterioration, while also enhancing the ability to predict success and improve the overall process.

Our study is not without limitations. First, we’ve studied a 60-mmHg PaO_2_ level at the end of AT as a threshold of aborting the test. This cutoff roughly represents an arterial saturation of 90%, which is higher that the AAN guideline for aborting the AT, set as 85%. As discussed, this cutoff results from our national directive for AT. Nevertheless, because of linear correlation and rapid oxygenation drops below these levels, setting this cutoff still reflects a safety conduct. Second, our study contains a limited group of patients, with a relatively small number of patients reaching the threshold for aborting AT. This limitation results from the database being a single-center experience. Perhaps a larger study by several centers could represent the population more accurately. Nevertheless, our database reflects an AT process done by a small group of physicians and thus is highly homogenic.

## Conclusions

PaO_2_ values at the end of AT are strongly correlated with PaO_2_ values at the beginning of the test. A cutoff value around 300 mmHg PaO_2_ at the beginning of the AT may aid in avoiding saturation drops below 90%.

## References

[1] De Georgia MA. History of brain death as death: 1968 to the present. J Crit Care. 2014;29:673–8. 24930367 10.1016/j.jcrc.2014.04.015

[2] Shewmon DA, Sylmar CA, Verheijde JL, Rady MY. Evidence-based guideline update: determining brain death in adults: report of the Quality Standards Subcommittee of the American Academy of Neurology. Neurology. 2011;76(3):308. 21302378

[3] Greer DM, Kirschen MP, Lewis A, Gronseth GS, Rae-Grant A, Ashwal S, et al. Pediatric and adult brain death/death by neurologic criteria consensus guideline. Neurology. 2023. 10.1212/WNL.0000000000207740. 37821233 10.1212/WNL.0000000000207740 PMC10791061

[4] Wijdicks EFM, Varelas PN, Gronseth GS, Greer DM. Evidence-based guideline update: determining brain death in adults: report of the Quality Standards Subcommittee of the American Academy of Neurology. Neurology. 2010;74:1911–8. 20530327 10.1212/WNL.0b013e3181e242a8

[5] Truog RD, Robinson WM. Role of brain death and the dead-donor rule in the ethics of organ transplantation. Crit Care Med. 2003;31:2391–6. 14501972 10.1097/01.CCM.0000090869.19410.3C

[6] Lewis A, Kirschen MP, Greer D. The 2023 AAN/AAP/CNS/SCCM pediatric and adult brain death/death by neurologic criteria consensus practice guideline: a comparison with the 2010 and 2011 guidelines. Neurol Clin Pract. 2023;13: e200189. 37829552 10.1212/CPJ.0000000000200189 PMC10567121

[7] Greer DM, Shemie SD, Lewis A, Torrance S, Varelas P, Goldenberg FD, et al. Determination of brain death/death by neurologic criteria: the World Brain Death Project. JAMA. 2020;324:1078–97. 32761206 10.1001/jama.2020.11586

[8] Spinello IM. Brain death determination. J Intensive Care Med. 2015;30:326–37. 24227449 10.1177/0885066613511053

[9] Sveen WN, Antommaria AHM, Gilene SJ, Stalets EL. Adverse events during apnea testing for the determination of death by neurologic criteria: a single-center, retrospective pediatric cohort. Pediatr Crit Care Med. 2023;24:399–405. 36815829 10.1097/PCC.0000000000003198

[10] Sayan HE. Retrospective analysis of the apnea test and ancillary test in determining brain death. Rev Bras Ter Intensiva. 2020;32:405–11. 33053030 10.5935/0103-507X.20200069 PMC7595719

[11] Busl KM, Lewis A, Varelas PN. Apnea testing for the determination of brain death: a systematic scoping review. Neurocrit Care. 2021;34:608–20. 32524528 10.1007/s12028-020-01015-0 PMC7286635

[12] Yee AH, Mandrekar J, Rabinstein AA, Wijdicks EFM. Predictors of apnea test failure during brain death determination. Neurocrit Care. 2010;12:352–5. 20217276 10.1007/s12028-010-9343-4

[13] Kim JJ, Kim EY. Identification of hemodynamic risk factors for apnea test failure during brain death determination. Transplant Proc. 2019;51:1655–60. 31255358 10.1016/j.transproceed.2019.04.029

